# Effects of labor induction in obesity with delayed pregnancy: A retrospective study based on Chinese obese primipara

**DOI:** 10.3389/fendo.2022.1055098

**Published:** 2023-01-11

**Authors:** Shuhua Liu, Bing Song, Dehong Liu, Chenmin Zheng, Xiumei Wu, Zhaolian Wei, Xianxia Chen

**Affiliations:** ^1^ Department of Obstetrics and Gynecology, Anhui Province Maternity and Child Health Hospital, Hefei, China; ^2^ Department of Obstetrics and Gynecology, The First Affiliated Hospital of Anhui Medical University, Hefei, China

**Keywords:** labor induction, body mass index, obese, primipara, delayed pregnancy

## Abstract

**Objective:**

To test the hypothesis that obese primiparous women with an unfavorable cervix in delayed pregnancy may experience a worse induction of labor.

**Study design:**

In total, 467 primiparas with poor cervical condition and delayed pregnancy (gestational age [GA]: >40weeks) were divided into an obese primiparas group (body mass index [BMI] >30kg/m^2^; n=166) and a non-obese primiparas group (BMI < 30kg/m^2^; (n=301). Labor was induced by various methods, double balloon, dinoprostone inserts, and amniotomy combined with oxytocin depending on the Bishop score. Experimental data were analyzed by Statistical Product Service Solutions (SPSS).

**Results:**

BMI in the obese primiparas group was higher than in the non-obese group (33.91 ± 2.67 versus 24.09 ± 5.78, p<0.001), and there were significant differences in uterine tone and duration of contractions between the two groups in the second stage of labour (p=0.041, p=0.026, respectively).The rate of cesarean section (CS) was significantly higher in the primiparas group (23.49% versus 12.29%; P=0.002). There was a significant difference between the two groups in terms of the duration of time to vaginal delivery (VD) (18h versus 8h; P <0.001) while the duration until VD in the obese primiparas group within 12 hours and 24 hours was significantly longer (P <0.001). After adjusting for possible confounders, caesarean section rates remained high in the obese primiparas women (OR: 2.564;95%CI1.919,3.864;P<0.001). Similarly, after adjusting for the same confounding factors, obese primiparas women increased the duration until VD within 24 h by 3.598 hours.

**Conclusion:**

Obese primiparas with an unfavorable cervix in delayed pregnancy have a significantly higher risk of CS and a longer duration until VD than non-obese primiparas during labor induction.

## Introduction

Over the past 30 years, the BMI of the general population has increased; thus, obesity has become a global problem (1, 2). A large meta-analysis of more than 100,000 subjects showed that almost 47% of pregnant women gained more weight than recommended (3). Furthermore, this previous study pointed out that obesity exerts multiple deleterious effects on reproduction and pregnancy outcomes, such as the increased incidence of gestational diabetes mellitus, hypertensive disorders of pregnancy, macrosomia, labor induction, dystocia and stillbirth (3–8). In addition, an increase in pre-pregnancy BMI has been associated with an increase in the rates of delivery by CS (9–15), overall delayed labor (11, 12, 16) and a higher rate of vaginal delivery failure (17, 18), thus requiring higher doses of oxytocin and longer exposure times to oxytocin (12, 14, 15, 19).

Obese pregnant women have become a worldwide concern and a challenge for obstetricians in clinical practice. Some studies have indicated that delayed pregnancy is more common among obese pregnant women (20, 21). However, few studies have focused on obese primiparas as the main research object with regards to labor induction in delayed pregnancy. Maged et al. investigated 129 primigravidas who had cesarean sections, including 17 cesarean sections in 64 obese patients and 15 cesarean sections in 65 non-obese patients, and found that the rates of cesarean section were comparable between the two groups (10). Furthermore, many previous studies on obese pregnant women did not separate primiparas from multiparous women, thus potentially resulting in bias. Studying obese multiparous and primiparous women together may not be an appropriate strategy. Previous studies have highlighted that the Bishop score is not a good indicator of successful labor induction and adverse maternal neonatal outcomes and complications in multiparous women, and noted that labor induction in multiparous women is safe and successful regardless of the initial Bishop score (22). Therefore, in this study, we only investigated obese primiparous women at the time of labor induction. Our aim was to test the hypothesis that obese primiparous women may experience a worse induction of labor. Our findings should guide Chinese clinicians to better manage the weight of obese pregnant women.

## Materials and methods

### Participants

The analysis of clinical data was performed using information from hospitalized patients at Anhui Province Maternity and Child Health Hospital in Hefei, China between March 2019 and April 2022. In total, 467 primiparas with poor cervical condition and delayed pregnancy (GA>40weeks) were divided into an obese primiparas group (BMI>30kg/m^2^; n=166) and a non-obese primiparas group (BMI< 30kg/m**^2^**; n=301). Other inclusion criteria were as follows: head presentation, singleton, normal fetal heart rate (FHR) and a Bishop score <6 points. We excluded gestational diabetes, gestational hypertension, cephalopelvic disproportion, macrosomia (> 4.5 kg), spontaneous labor onset. According to the principles of the Declaration of Helsinki, the Ethical Review Board of Anhui Province Maternity and Child Health Hospital approved this research (Ethics number: YYLL-2022-04-FA-01).

### Study design

According to the Bishop score, as assigned by an experienced obstetrician, the methods of labor induction included double balloon, dinoprostone insert, and amniotomy combined with oxytocin. A double balloon (Cook Medical, USA) was placed at 8pm and removed at 8am the following morning. If the patient had no contractions, then amniotomy was performed. FHR was observed for 30 min; then, we commenced oxytocin (H32025280, Nanjing Xinbai Pharmaceutical Co., Ltd. Co., Ltd., China) to induce efficient uterine contractions (3-4 contractions of 200 Montevideo units over a 10 min period). Dinoprostone (Propess, Switzerland) was placed into the posterior fornix of the vagina; contractions and FHR were monitored during placement. The drug was withdrawn in the case of excessive or too frequent contractions or a Bishop score greater than 6 points. Oxytocin was used when the uterus did not contract effectively, or labor progressed slowly. If the Bishop approached 6 points, then we performed amniotomy combined with oxytocin.

### Statistical analysis

Clinical data were analyzed by SPSS software (SPSS Inc, Chicago, IL,version 24.0). Data that conformed to a normal distribution are represented by mean ± standard deviation (SD). The T-test, Chi-squared test and Fisher’s exact test were used to test for differences between normally distributed data and differences in percentages or proportions between two or more groups of data, respectively. Median was used for non-normally distributed data. Logistic regression analysis and multivariable regression are used to analyze data relating to mode of delivery for obese vs non-obese groups and the duration until VD within 24 h for obese vs non-obese groups, respectively. GraphPad Prism version 9.0 for Windows(GraphPad Software, San Diego, California USA, www.graphpad.com) is used to forest plot. P < 0.05 was considered as a significant difference.

## Results

**Figure 1:**
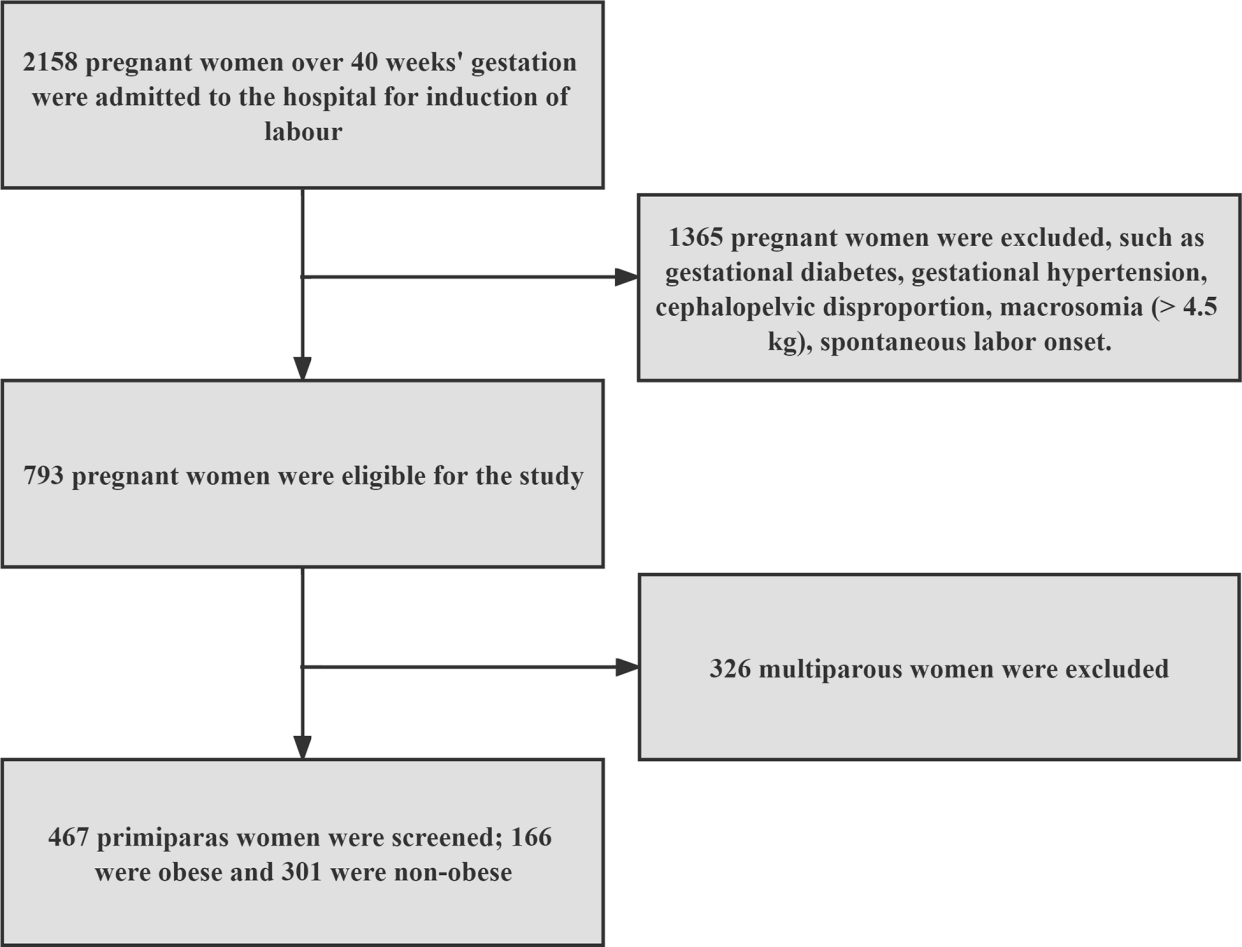
Flowchart of the research object.

**Figure 1** shows that we ultimately screened 467 eligible nulliparous women with poor cervical condition and delayed pregnancy for the study, divided into obese group (n=166) and non-obese group (n=301).

### Basic clinical data

**Table 1** shows the general clinical data related to 166 obese primiparas and 301 non-obese primiparas. There was a significant difference in BMI between the two groups (33.91 ± 2.67 versus 24.09 ± 5.78; P<0.001),and there were significant differences in uterine tone and duration of contractions between the two groups in the second stage of labour (p=0.041, p=0.026, respectively).Maternal age, GA, Bishop score, amniotic fluid index (AFI), and the proportion of cases of epidural analgesia were not significantly different when compared between the two groups (all P>0.05). Educational background (junior high school, high school, university), birth weight (>4000 g, fetal growth restriction [FGR], 2500–4000 g) and induction method (double-balloon, dinoprostone insert, amniotomy combined with oxytocin) were not significantly different when compared between the two groups (P>0.05).

**Table 1:** Basic clinical data.

Parameter	Obese primiparas group (n=166)	Non-obese primiparas group (n=301)	*P*
Maternal age (years)	29.03 ± 4.26	28.92 ± 4.36	*0.801*
**BMI** (kg/m^2^)	33.91 ± 2.67	24.09 ± 5.78	***<0.001***
**GA**(weeks)	40.29 ± 0.13	40.30 ± 0.14	*0.627*
**Bishop score**	4 (2,5)	4 (2,5)	*0.594*
**AFI**(mm)	125 (48,180)	125 (55,165)	*0.443*
Educational background			
Junior high school (n)	28	45	*0.807*
High school (n)	25	50	
College/University (n)	113	206	
**Birth weight (g)**	3404.9 ± 334.4	3370.5 ± 347.7	*0.300*
Birth weight>4000 g (n)	15	14	*0.178*
FGR (n)	6	10	
Birth weight 2500-4000g(n)	145	277	
Induction method			
Double-balloon (n)	82	147	*0.147*
Dinoprostone insert (n)	70	130	
Amniotomy combined with oxytocin (n)	14	24	
First stage of labor			
Uterine tone(mmHg)	60.35 ± 17.93	61.26 ± 17.12	*0.678*
Contraction frequency(min)	3.72 ± 0.78	3.68 ± 0.85	*0.701*
Duration of contractions(s)	32.11 ± 4.12	33.21 ± 3.69	*0.783*
Second stage of labor			
Uterine tone(mmHg)	65.25 ± 17.11	73.36 ± 16.85	***0.041***
Contraction frequency(min)	3.35 ± 0.65	3.24 ± 0.78	*0.138*
Duration of contractions(s)	33.24 ± 4.65	38.56 ± 3.85	***0.026***
Third stage of labor			
Uterine tone(mmHg)	86.23 ± 16.52	89.56 ± 15.69	*0.569*
Contraction frequency(min)	2.35 ± 0.75	2.21 ± 0.86	*0.678*
Duration of contractions(s)	57.45 ± 5.12	59.62 ± 4.98	*0.812*
**Epidural analgesia**	134/166	241/301	*0.904*

AFI, amniotic fluid index; BMI, body mass index; GA, gestational age; FGR, fetal growth restriction; g, gram; min, minute; s, second; mm, millimeter.

Bold values was statistically significant (P<0.05).

### Induction of labor

**Table 2** shows that the obese primiparas group had a higher rate of CS and a lower rate of VD when compared to the non-obese group (23.49% *versus* 12.29%; 76.51% *versus* 87.71%; P=0.002). In addition, the obese group had a longer duration until VD [median (range): 18 (2,40) *versus* 8 (2,41); P<0.001]. The obese primiparas group had a higher rate of CS delivery and a lower rate of VD than the non-obese primiparas group (P=0.002). Indications for CS were not significantly different between the two groups; nor was the time from the onset of induction to the decision to perform CS. In **Table 3**, adverse maternal and pediatric outcomes of delivery, including postpartum hemorrhage (PPH), emergency delivery (ED) and admission to the Neonatal Intensive Care Unit (NICU) were not significantly different when compared between the two groups (all P>0.05).

**Table 2:** The results of labor induction and delivery.

	Obese primiparas group (n=166)	Non-obese primiparas group (n=301)	*P*
Mode of delivery			
VD (n,%)	127/166 (76.51)	264/301 (87.71)	***0.002***
CS (n,%)	39/166 (23.49)	37/301 (12.29)	
Indications for CS (n,%)			
Failed induction	14/39 (35.90)	13/37 (35.14)	*0.791*
Failure of progress	11/39 (28.21)	14/37 (37.84)	
Fetal distress	9/39 (23.08)	7/37 (18.92)	
CDMR	5/39 (7.25)	3/37 (8.11)	
**Duration until VD (hour)**	18 (2,40)	8 (2,41)	***<0.001***
<12hours (n,%)	50/127 (39.37)	172/264 (65.15)	***<0.001***
≤ 24hours (n,%)	62/127 (48.82)	205/264 (77.65)	***<0.001***
Delivery time for CS			
≤ 12hours (n,%)	14/39 (35.90)	15/37 (40.54)	*0.814*
≤ 24hours (n,%)	25/39 (64.10)	24/37 (64.86)	*0.446*

VD, Vaginal delivery; CS, Cesarean section; CDMR, cesarean delivery on maternal request; Bold values was statistically significant (P<0.05).

**Table 3:** Adverse maternal and child outcomes of delivery.

	Obese primiparas group (n=166)	Non-obese primiparas group (n=301)	*P*
PPH (n,%)	6/166 (3.61)	18/301 (5.98)	*0.227*
ED (n,%)	2/127 (1.57)	16/264 (6.06)	*0.068*
Admission to NICU (n,%)	8/166 (4.82)	7/301 (2.33)	*0.172*

PPH, post-partum hemorrhage; ED, Emergency delivery; NICU, Neonatal Intensive Care Unit.

### Factor analysis for the mode of delivery and duration until VD within 24 h for obese vs non-obese groups

**Figure 2:**
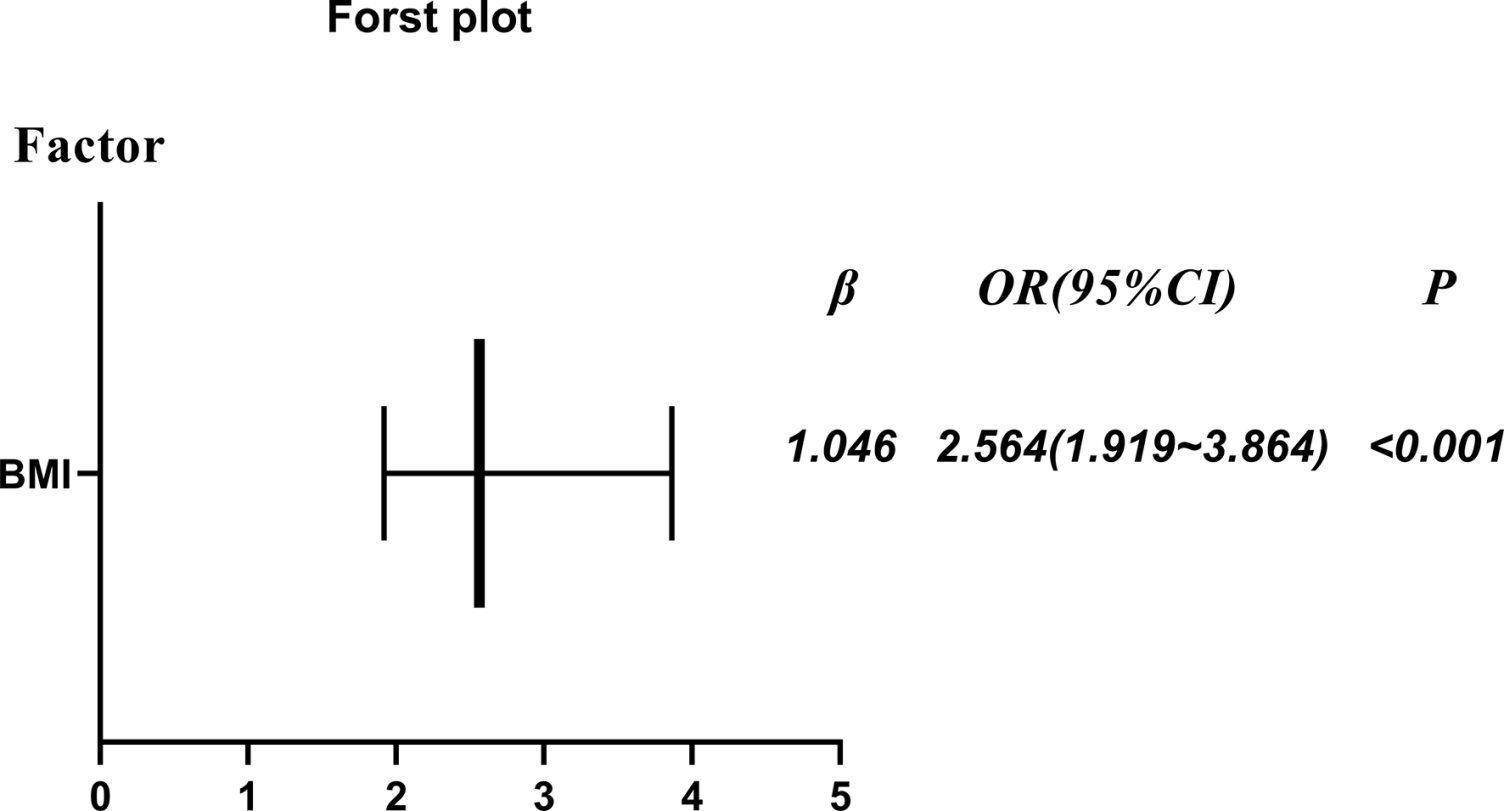
The rate of cesarean section (obese primiparous versus non-obese primiparous group) after adjustment for maternal age, GA, BMI, epidural analgesia, bishop score, birth weight and AFI.

In **Figure 2**, after adjustment for maternal age, GA, BMI, epidural analgesia, bishop score, birth weight and AFI, the rate CS was still higher in the obese group than the non-obese group (P<0.001; odds ratio [OR] = 2.546; 95% confidence interval [CI] = 1.919-3.864). In addition, after adjusting for the same factors, obesity had the risk of increasing duration until VD within 24 h by 3.589 h (P<0.001; β=3.589; 95% CI = 3.891-4.986) in **Table 4**.

**Table 4:** Multivariable regression for duration until VD within 24 h for obese vs non-obese groups.

Factors	β	95%CI		*P*
		Down	Up	
Duration until VD within 24 h(obese primiparas group vs non-obese primiparas group)	3.589	3.891	4.986	***<0.001***

β coefficients is duration until VD within 24 h after adjustment for maternal age, GA, BMI, epidural analgesia, bishop score, birth weight and AFI.

Bold values was statistically significant (P<0.05).

## Discussion

In China, termination of pregnancy can be considered after 40 weeks’ gestation if the pregnant woman wishes to terminate the pregnancy, and in the case of pregnancy complications, the timing of termination may be earlier according to the guidelines. The number of obese pregnant women has gradually increased over recent years in China. Although many studies have demonstrated a close association between female obesity and delayed pregnancy (20), few studies have investigated the outcome of obese primiparas with an unfavorable cervix with regards to labor induction in prolonged pregnancy. Compared with non-obese patients, obese patients tend to adopt cesarean delivery earlier (9). In this study, after adjusting for possible confounding factors, the caesarean section rate remained higher among obese primiparous women than in non-obese primiparas women (OR: 2.564;95%CI1.919,3.864;P<0.001).This result suggests that obesity is at higher risk of induction failure in obesity with delayed pregnancy. This result has also been shown in previous studies that the obese pregnant women had a higher rate of failure to induce labor when compared with the non-obese group (OR=2.02) (10). However, their subjects included obese nulliparous women and obese multiparous women. Taking into account the results of previous studies, the outcome of labor induction in multiparous women was not affected by the cervical Bishop score (22). Therefore, we applied an improved experimental design and selected obese primiparas with an unfavorable cervix in delayed pregnancy as study subjects. Although, after adjusting for the same confounding factors, obese Primiparas women still increased the duration until VD within 24 h by 3.598 hours. Similar results were reported in the results of Nuthalapaty (11).Their study showed that for every 10kg increase in maternal weight from the initiation of oxytocin until the delivery of the fetus, the duration of labour increased by 30 minutes (11).This suggests that obstetricians should pay more attention to the management of optimal weight gain in obese pregnant women to improve the rate of VD and reduce the incidence of maternal and infant complications.

The pathophysiological mechanisms underlying the effects of obesity on childbirth remain unclear. Some studies have suggested that endocrine factors, oxytocin receptor sensitivity, and reduced uterine contractility may all be factors responsible for prolonged labor and higher CS rates in obese patients (8, 23–25). In addition, studies have shown that obese women were more likely to have a low Bishop score (26, 27),which may be related to hormonal imbalance caused by excessive fat accumulation, delayed natural labor and an immature cervix (23, 28).Carvajal et al. reported that the myometrium of obese pregnant women exhibited lower expression levels of connexin 43, reduced lower oxytocin receptor functionality and higher potassium channel activity. Obese women are known to exhibit higher concentrations of adipokines which may reduce muscle contractility by inhibiting the RhoA/ROCK pathway in the muscle layer. The high cholesterol levels of obese pregnant women are known to alter the myometrial membranes, especially the pits, thus inhibiting the functionality of oxytocin receptors and increasing the activity of K^+^ channels (25). Changes in myometrial cells and their local environment may reduce myometrial contractility; this could explain the delayed labor and increased rates of CS delivery in obese pregnant women, at least in part (25).In addition, studies have pointed out that the resistance of leptin in obese patients leads to an increase in leptin concentration, resulting in a decrease in uterine contractility (29). And leptin also inhibits uterine contractility *in vitro* (30).In addition, studies have pointed out that the resistance of leptin in obese patients leads to an increase in leptin concentration, resulting in a decrease in uterine contractility. And leptin also inhibits uterine contractility *in vitro*. Since this study is a retrospective analysis, in China, leptin is not used as a routine prenatal examination item during pregnancy, we cannot know the relevant content, these previous studies make us to improve the prospective experimental research in the future to reveal the relevant mechanism.

Obesity is associated with increased neonatal morbidity, which does not change with prolonged second stage of labour (31). In a previous study, Ellekjaer et al. found that the incidence of a PPH > 1000ml increased with increasing BMI during the first trimester (9). However, in the present study, we found that adverse maternal and pediatric outcomes, such as PPH, ED and NICU admission, were not significantly different when compared between the two groups. Moreover, there were no significant differences between the two groups with regards to indications for CS delivery, such as fetal distress which may increase the need for neonatal rescue and admission to the NICU. Maged et al. reported similar, with comparable rates of PPH between the two study populations (10).

It is also reasonable that we assess BMI directly during induction of labour. Because true obesity should have an impact on the process of childbirth. Previous studies have suggested that the amount of weight gain during pregnancy is an important predictor of maternal and infant outcomes. However, there are some limitations to our study that need to be considered. Our hospital is a tertiary hospital for women and children and is not responsible for the creation of health manuals. Some patients do not establish a health care manual at a community hospital until more than 12 weeks of gestation. Unfortunately, some of the data collected were from health care manuals with incomplete preconception body mass index, and some of the data were biased by patient recall. These data can enrich our study by assessing the correlation between gestational weight gain and our findings. But our study also had several key advantages, including a relatively rich data dataset, careful delivery management, birth canal examination and Bishop scoring by experienced clinicians, tight control of CS indications, and rigorous inclusion criteria and detailed statistical analysis.

## Conclusions

Obese primiparas with an unfavorable cervix in delayed pregnancy have higher risks of CS and a longer duration until VD than non-obese primiparas during the induction of labor. Therefore, there is a need to manage weight gain during pregnancy to improve the success rate of labor induction.

## Data availability statement

The original contributions presented in the study are included in the article/supplementary material. Further inquiries can be directed to the corresponding authors.

## Ethics statement

The studies involving human participants were reviewed and approved by the Ethical Review Board of Anhui Province Maternity and Child Health Hospital. The patients/participants provided their written informed consent to participate in this study.

## Author contributions

SL contributed to conception and design. DL, CZ, and XW developed the methodology. SL collected and analyzed the data. SL and BS wrote, reviewed and revised the manuscript. XC and ZW contributed to administrative, technical and material support. All authors read and approved the final manuscript.
